# Protective effects of allicin against stanozolol‐induced cardiotoxicity: Physiological and histopathological evidence in a rabbit model

**DOI:** 10.1002/ame2.70035

**Published:** 2025-06-18

**Authors:** Mohammed Hayder Asker, Noor AL‐Huda Salah AL‐Zuhairy, Wassan Mhammed Husain, Mustafa Riyadh Abdullah

**Affiliations:** ^1^ Department of Pharmacology and Toxicology, College of Pharmacy Mustansiriyah University Baghdad Baghdad Iraq; ^2^ Medical College Ibn Sina University of Medical and Pharmaceutical Sciences Baghdad Iraq

**Keywords:** anabolic steroids, antioxidants, cardiovascular diseases, oxidative stress, rabbit model

## Abstract

**Background:**

There are many forms of anabolic steroids, including stanozolol (Winstrol), which are popular for their muscle‐building effects but dangerous to the heart. This present work is aimed at evaluating the pharmacologica impact of allicin, a natural attribute obtained from garlic, on obstructing cardiac injury in rabbits that received stanozolol.

**Methods:**

Thirty rabbits were divided into three groups: control, stanozolol‐treated, and stanozolol plus allicin. Cardiac function was assessed by measuring troponin, creatine kinase (CK), Galectin‐3, and GDF‐15. Oxidative stress and antioxidant markers, including malondialdehyde (MDA), glutathione, and catalase, were analyzed. Inflammatory mediators such as C‐reactive protein (CRP), interleukin‐6 (IL‐6), NF‐κB, iNOS, nitric oxide (NO), tumor necrosis factor‐alpha (TNF‐α), and interleukin‐1 beta (IL‐1β) were evaluated. Lipid profile parameters, including total cholesterol, low‐density lipoprotein (LDL), and high‐density lipoprotein (HDL), were measured. Histopathological examination assessed myocardial damage, fibrosis, and collagen deposition.

**Results:**

Stanozolol administration significantly increased cardiac damage markers, oxidative stress, and inflammatory mediators while causing dyslipidemia, characterized by elevated LDL and total cholesterol and reduced HDL. Allicin co‐administration effectively countered these effects by reducing oxidative stress and inflammation, restoring antioxidant balance, and improving lipid profiles. Histopathological analysis revealed severe myocardial disorganization, necrosis, and fibrosis in the stanozolol group, whereas the allicin‐treated group exhibited preserved myocardial structure with reduced collagen deposition.

**Conclusion:**

Allicin significantly mitigates stanozolol‐induced cardiotoxicity by reducing oxidative stress, inflammation, lipid dysregulation, and myocardial damage, as evidenced by biochemical and histopathological findings. These results suggest that allicin may serve as a potential therapeutic agent to counteract the cardiovascular risks associated with anabolic steroid use.

## Graphical abstract

There are many forms of anabolic steroids, including stanzolo (Winstrol), which are popular for their muscle‐building effects but dangerous to the heart. This present work is aimed at evaluating the pharmacological impact of allacin, a natural attribute obtained from garlic on obstructing cardiac injury in rabbits that received stanzolo. A total of 30 rabbits were divided into three groups: control, stanzolo‐treated and Stanzolo plus Allacin. Chemical parameters comprised of C‐reactive protein (CRP) serum, malondialdehyde (MDA), glutathione, troponin and cardiac enzymes were measured biochemically to test the cardiac dysfunction and oxidative stress after 30 days of practice. Another investigation carried out in relation to the structural cardiac changes involved histopathological evaluation. Findings revealed that, relative to placebo, stanzolo upregulated the levels of inflammation marker CRP, lipid peroxidation marker MDA, and cardiac troponin, and ‘downregulated the antioxidant glutathione’. On the other hand, rabbits in the allacin‐treated group had lower levels of these markers, which meant a possible cardioprotection against exogenous steroids. Biochemical results were also for the most part echoed by histopathological changes which revealed better preserved myofiber structure and less myocardial damage in allacin treated animals compared with stanzolo treated rats. The current research highlights allacin as a possible protective agent against anabolic steroid induced cardiovascular toxicity, offering important findings about therapeutic approach in steroid consumers.

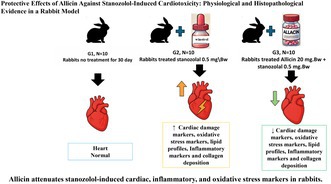

## 1 INTRODUCTION

Stanzolo, or Winstrol, is an anabolic steroid that belongs to the group of drugs that have a hormonal structure and is based on testosterone, which is useful in the practice of sports medicine due to the given effect. Though they can enhance muscular development and ramp up exercise capacity, they are linked to various side effects most relating to the cardiovascular system. An amount of literature is available on the cardiotoxic effect of anabolic steroids in various population groups and has pointed to the anabolic steroids' ability to cause myocardial injury, dyslipidemia, and changes in speckle tracking‐based measures of cardiac function.^1^, ^2^ Some of the processes that explain these adverse effects cover the following: increased levels of oxidative stress and inflammation and altered guidelines of cardiac biomarkers, which affect the overall integrity and function of the cardiac tissues.^3^, ^4^

It has been postulated that oxidative stress is the main link in the causal chain of steroid‐induced cardiotoxicity. Reactive oxygen species (ROS) production that is closely associated with lipid peroxidation and myocardial damage has also been implicated with anabolic steroids.^5^ The oxidative stress in addition to adversely affecting cellular functioning also activates inflammatory responses, which in turn makes a worse cardiac damage.^6^ Therefore, the present study hypothesized that there are increased systemic inflammations as shown by the levels of C‐reactive protein (CRP) and interleukin‐6 (IL‐6) that can worsen cardiovascular outcomes.^7^

Due to the side effects the anabolic steroids produce on the cardiovascular system different protective approaches have been investigated to power anabolic steroids including the use of natural compounds with scouring antioxidant and anti‐inflammatory properties. It has a role in antioxidant activities and cardiovascular health, a fact which has made such substances as Allicin found in garlic gain attention for cardioprotective properties.^8^, ^9^ Earlier trials have shown that allicin could decrease levels of oxidative diseases and dysfunction of the endothelial layer, which implies the possibility of using this agent in treating cardiovascular pathology.^10^

The purpose of this present work is to determine the beneficial physiological role of allicin in protecting rabbits subjected to stanozol‐induced cardiotoxicity. In addition, efforts will be made to relate these biochemical changes to histopathological alterations in cardiac tissue. Thus, the present study aims to cast light on the ability of allicin to prevent anabolic steroid‐elicited cardiotoxicity.

## 2 METHODS

### 2.1 Animal preparation and experimental design

A total of 30 adult male rabbits, weighing approximately 2.5 to 3.0 kg, were housed under controlled conditions with a 12‐h light–dark cycle and ad libitum access to food and water. The experimental animals received two weeks to acclimate before the study initiation. The protocol received approval from the Institutional Animal Care and Use Committee of [Mustansiriyah University] under reference number [No: 42 on 1/9/2024] to ensure ethical guidelines protecting animal well‐being.

Research subjects received distribution across three separate groups:

- Control Group (*n* = 10): Rabbits received no treatment or received a placebo injection.
- Stanozolol Group (*n* = 10): Rabbits were treated with Stanozolol (Winstrol) at a dosage of 0.5 mg/kg body weight for 30 days.^11^
- Allicin + Stanozolol Group (*n* = 10): Rabbits received a 0.5 mg/kg body weight Stanozolol (Winstrol) dosage together with a 20 mg/kg body weight allicin dose for 30 days according to Shaikh.^12^

### 2.2 Biochemical marker analysis

Blood samples were collected from each rabbit at baseline and the end of the treatment period for biochemical analyses. Blood samples were collected from each animal via the jugular vein. Blood samples were centrifuged at 3000 revolutions per minute (rpm) for 15 min, and serum was collected and stored at −20°C until analysis. The following biomarkers were analyzed.

#### 2.2.1 Cardiac damage markers

Troponin and Creatinine Kinase (CK) levels were measured using enzyme‐linked immunosorbent assay (ELISA) kits (abcam, ab200016, ab285275) as per the manufacturer's instructions.

Galectin‐3 and Growth Differentiation Factor‐15 (GDF‐15) levels were quantified using specific ELISA assays (E‐EL‐H1470, RayBio®).

#### 2.2.2 Oxidative stress and antioxidant markers

Levels of MDA, Glutathione, and Catalase were assessed using colorimetric methods. MDA levels were measured based on the thiobarbituric acid reactive substances (TBARS) assay (Solarbio Science & Technology Co., Ltd).

#### 2.2.3 Lipid profile

Total cholesterol, Low‐Density Lipoprotein (LDL), and High‐Density Lipoprotein (HDL) levels were analyzed using enzymatic methods and commercially available kits (LINEAR CHEMICALS S.L.).

#### 2.2.4 Inflammatory markers

CRP, IL‐6, procalcitonin, and aspartate aminotransferase (AST) were assessed using ELISA kits according to the manufacturer's protocols (Abcam, ab260058, ab178013, ab100630, ab263881).

#### 2.2.5 Damage markers

Blood samples were collected and processed to evaluate damage markers, including NF‐κB, iNOS, NO, Bcl‐2, Caspase‐3, TNF‐α, and IL‐1β. Enzyme‐linked immunosorbent assay (ELISA) was used to quantify NF‐κB (ab176648—Abcam), Bcl‐2 (ab119506—Abcam), Caspase‐3 (# BMS2012INST—Thermo Fisher Scientific Inc), TNF‐α (# KHC3011—Thermo Fisher Scientific Inc), and IL‐1β levels (# KHC0011—Thermo Fisher Scientific Inc), while iNOS activity and NO concentration were measured using spectrophotometric assays (AB5384—Millipore).

### 2.3 Histopathological examination

Following biochemical analysis, all animals were euthanized by an overdose of anesthesia (ketamine/xylazine), and heart tissues were harvested. Samples were fixed in 10% buffered formalin for 24–48 h, dehydrated through graded alcohols, embedded in paraffin, and sectioned at 4–5 μm thickness.

The scoring system parameters,

Each parameter was scored on a scale of 0 to 3, as follows:

0: No detectable abnormality.
1: Mild changes (e.g., scattered inflammatory cells or slight disarray).
2: Moderate changes (e.g., focal necrosis or moderate fibrosis).
3: Severe changes (e.g., extensive necrosis or widespread fibrosis).

The complete measure of cardiovascular tissue injury used the combination of scores from all evaluation criteria to assess the extent of myocardial damage.^13^

Left ventricle collagen deposition received assessment using biomedical and histological techniques. The histology examination required heart tissue fixation through 10% neutral‐buffered formalin while the paraffin embedding was followed by sectioning at 5 μm thickness. Masson's trichrome staining allowed researchers to view collagen fibers in cardiac tissue specimens.

### 2.4 Relative heart weight

The relative heart weight (RHW) value was calculated as follows equation^14^:

$$\mathrm{RHW}=\text{Heart Weight}\;\left(g\right)/\text{Body Weight}\;\left(\mathrm{kg}\right)$$

### 2.5 Statistical analysis

The research data received analysis through Statistical Package for the Social Sciences (SPSS version 26). All variables received descriptive statistical analysis and one‐way ANOVA provided group mean comparison and analysis. The data analysis conducted a Tukey's HSD test for post hoc evaluation. Results with a statistical significance at *p* < 0.05 were determined to be valid.

## 3 RESULTS

### 3.1 Cardiac damage markers

The three experimental groups had distinct troponin and CK levels along with Galectin‐3 and GDF‐15 levels which indicate different extents of cardiac injury. Compared to the control group, the stanozolol‐only group exhibited significantly elevated levels of Troponin (0.5 ± 0.15 ng/mL) and CK (300 ± 20 U/L), indicative of cardiotoxicity due to stanozolol administration. Allicin treatment in the Stanozolol + Allicin group mitigated these elevations, with Troponin and CK levels reduced to 0.2 ± 0.1 ng/mL and 150 ± 15 U/L, respectively. This reduction was statistically significant, with *p*‐values <0.005 for Troponin and 0.002 for CK (Table 1).

**TABLE 1:** Cardiac damage markers in different experimental groups.

Marker	Control group	Stanozolol‐only group	Stanozolol + Allicin group	*p*‐Value (control vs. stanozolol)	*p*‐Value (stanozolol vs. allicin)
Troponin (ng/mL)	0.02 ± 0.01	0.5 ± 0.15	0.2 ± 0.1	<0.001	0.005
Creatinine kinase (U/L)	75 ± 10	300 ± 20	150 ± 15	<0.001	0.002
Galectin‐3 (ng/mL)	5 ± 1	15 ± 3	8 ± 2	<0.001	0.01
GDF‐15 (pg/mL)	500 ± 50	2000 ± 150	1200 ± 100	<0.001	0.01

### 3.2 Oxidative stress and antioxidant defense markers

Markers of oxidative stress and antioxidant defense, including MDA, Glutathione, and Catalase, showed significant differences among the experimental groups. MDA levels increased from 1 ± 0.2 nmol/mL in the control group to 4 ± 0.5 nmol/mL in the stanozolol‐only group, while the Stanozolol + Allicin group showed a reduction to 2 ± 0.3 nmol/mL (*p* < 0.001, *p* = 0.003). Glutathione levels decreased from 9 ± 1 μmol/L in the control group to 4 ± 1 μmol/L in the stanozolol‐only group, with the Stanozolol + Allicin group showing levels of 8 ± 1 μmol/L (*p* < 0.001, *p* = 0.01). Catalase activity dropped from 50 ± 5 U/mg in the control group to 20 ± 3 U/mg in the stanozolol‐only group, with an increase to 40 ± 5 U/mg in the Stanozolol + Allicin group (*p* < 0.001, *p* = 0.005) (Table 2).

**TABLE 2:** Oxidative stress and antioxidant defense markers.

Marker	Control group	Stanozolol‐only group	Stanozolol + Allicin group	*p*‐Value (control vs. stanozolol)	*p*‐Value (stanozolol vs. allicin)
MDA (nmol/mL)	1 ± 0.2	4 ± 0.5	2 ± 0.3	<0.001	0.003
Glutathione (μmol/L)	9 ± 1	4 ± 1	8 ± 1	<0.001	0.01
Catalase (U/mg)	50 ± 5	20 ± 3	40 ± 5	<0.001	0.005

### 3.3 Lipid profile

Increased Total Cholesterol and LDL levels in the stanozolol‐only group (120 ± 10 mg/dL and 70 ± 5 mg/dL, respectively) compared to controls reflect lipid dysregulation due to stanozolol. Allicin co‐administration resulted in significant improvements, reducing Total Cholesterol to 90 ± 8 mg/dL and LDL to 50 ± 4 mg/dL (*p*‐values <0.01 and 0.005, respectively), HDL levels, which were reduced in the stanozolol‐only group (25 ± 3 mg/dL) compared to controls (40 ± 5 mg/dL, *p* < 0.001), increased to 35 ± 4 mg/dL in the Stanozolol + Allicin group (*p* = 0.01), as seen in Table 3.

**TABLE 3:** Lipid profile markers.

Marker	Control group	Stanozolol‐only group	Stanozolol + Allicin group	*p*‐Value (control vs. stanozolol)	*p*‐Value (stanozolol vs. allicin)
Total cholesterol (mg/dL)	70 ± 5	120 ± 10	90 ± 8	<0.001	0.01
LDL (mg/dL)	35 ± 3	70 ± 5	50 ± 4	<0.001	0.005
HDL (mg/dL)	40 ± 5	25 ± 3	35 ± 4	<0.001	0.01

### 3.4 Inflammatory and organ damage markers

Markers of inflammation and organ damage, including CRP, IL‐6, Procalcitonin, AST, and Cystatin C, showed significant changes across experimental groups. CRP levels increased from 0.5 ± 0.1 mg/L in the control group to 5 ± 0.5 mg/L in the stanozolol‐only group, with a reduction to 2 ± 0.3 mg/L in the Stanozolol + Allicin group (*p* < 0.001, *p* = 0.01). Similarly, IL‐6 levels rose from 2 ± 0.5 pg/mL in the control group to 15 ± 2 pg/mL in the stanozolol‐only group and decreased to 7 ± 1 pg/mL with allicin co‐administration (*p* < 0.001, *p* = 0.005). Procalcitonin increased from 0.05 ± 0.01 ng/mL in the control group to 0.5 ± 0.05 ng/mL in the stanozolol‐only group, with a reduction to 0.2 ± 0.03 ng/mL in the Stanozolol + Allicin group (*p* < 0.001, *p* = 0.01). AST levels rose from 15 ± 2 U/L in the control group to 40 ± 5 U/L in the stanozolol‐only group and were reduced to 25 ± 4 U/L with allicin treatment (*p* < 0.001, *p* = 0.005). Lastly, Cystatin C levels increased from 0.6 ± 0.05 mg/L in the control group to 1.2 ± 0.1 mg/L in the stanozolol‐only group and decreased to 0.8 ± 0.08 mg/L in the Stanozolol + Allicin group (*p* < 0.001, *p* = 0.01). (Table 4).

**TABLE 4:** Inflammatory and organ damage markers ‐ general biomarkers.

Marker	Control group	Stanozolol‐only group	Stanozolol + Allicin group	*p*‐Value (control vs. stanozolol)	*p*‐Value (stanozolol vs. allicin)
CRP (mg/L)	0.5 ± 0.1	5 ± 0.5	2 ± 0.3	<0.001	0.01
IL‐6 (pg/mL)	2 ± 0.5	15 ± 2	7 ± 1	<0.001	0.005
Procalcitonin (ng/mL)	0.05 ± 0.01	0.5 ± 0.05	0.2 ± 0.03	<0.001	0.01
AST (U/L)	15 ± 2	40 ± 5	25 ± 4	<0.001	0.005
Cystatin C (mg/L)	0.6 ± 0.05	1.2 ± 0.1	0.8 ± 0.08	<0.001	0.01

Other Markers of inflammation and organ damage, showed in the control group, NF‐κB levels were 2 ± 0.5 pg/mL, while the Stanozolol‐only group showed a significant increase to 12 ± 2 pg/mL (*p* < 0.001), which was reduced to 6 ± 1 pg/mL in the Stanozolol + Allicin group (*p* = 0.005). Similarly, iNOS levels were 0.8 ± 0.1 U/L in the control group, rising to 3.5 ± 0.4 U/L in the Stanozolol‐only group (*p* < 0.001), and decreasing to 1.5 ± 0.3 U/L with Allicin treatment (*p* = 0.01). NO concentration followed the same trend, increasing from 15 ± 2 μmol/L in the control group to 50 ± 5 μmol/L with Stanozolol (*p* < 0.001) and decreasing to 30 ± 4 μmol/L with Allicin (*p* = 0.01). Bcl‐2 levels were significantly reduced in the Stanozolol‐only group (8 ± 1 ng/mL) compared to the control (20 ± 3 ng/mL, *p* < 0.001), but Allicin treatment restored it to 15 ± 2 ng/mL (*p* = 0.005). Caspase‐3 levels increased from 0.2 ± 0.05 ng/mL in the control to 1.2 ± 0.2 ng/mL with Stanozolol (*p* < 0.001), decreasing to 0.6 ± 0.1 ng/mL with Allicin (*p* = 0.005). TNF‐α levels showed a similar pattern, rising from 5 ± 1 pg/mL in the control to 18 ± 2 pg/mL with Stanozolol (*p* < 0.001) and decreasing to 10 ± 1 pg/mL with Allicin (*p* = 0.005). Finally, IL‐1β levels were 3 ± 0.5 pg/mL in the control, significantly increasing to 14 ± 1.5 pg/mL with Stanozolol (*p* < 0.001) before being reduced to 7 ± 1 pg/mL with Allicin treatment (*p* = 0.005). (Table 5).

**TABLE 5:** Inflammatory and organ damage markers ‐ cellular indicators.

Marker	Control group	Stanozolol‐only group	Stanozolol + Allicin group	*p*‐Value (control vs. stanozolol)	*p*‐Value (stanozolol vs. allicin)
NF‐κB (pg/mL)	2 ± 0.5	12 ± 2	6 ± 1	<0.001	0.005
iNOS (U/L)	0.8 ± 0.1	3.5 ± 0.4	1.5 ± 0.3	<0.001	0.01
NO (μmol/L)	15 ± 2	50 ± 5	30 ± 4	<0.001	0.01
Bcl‐2 (ng/mL)	20 ± 3	8 ± 1	15 ± 2	<0.001	0.005
Caspase‐3 (ng/mL)	0.2 ± 0.05	1.2 ± 0.2	0.6 ± 0.1	<0.001	0.005
TNF‐α (pg/mL)	5 ± 1	18 ± 2	10 ± 1	<0.001	0.005
IL‐1β (pg/mL)	3 ± 0.5	14 ± 1.5	7 ± 1	<0.001	0.005

### 3.5 Histopathological results

#### 3.5.1 Control group histopathology

**FIGURE 1:**
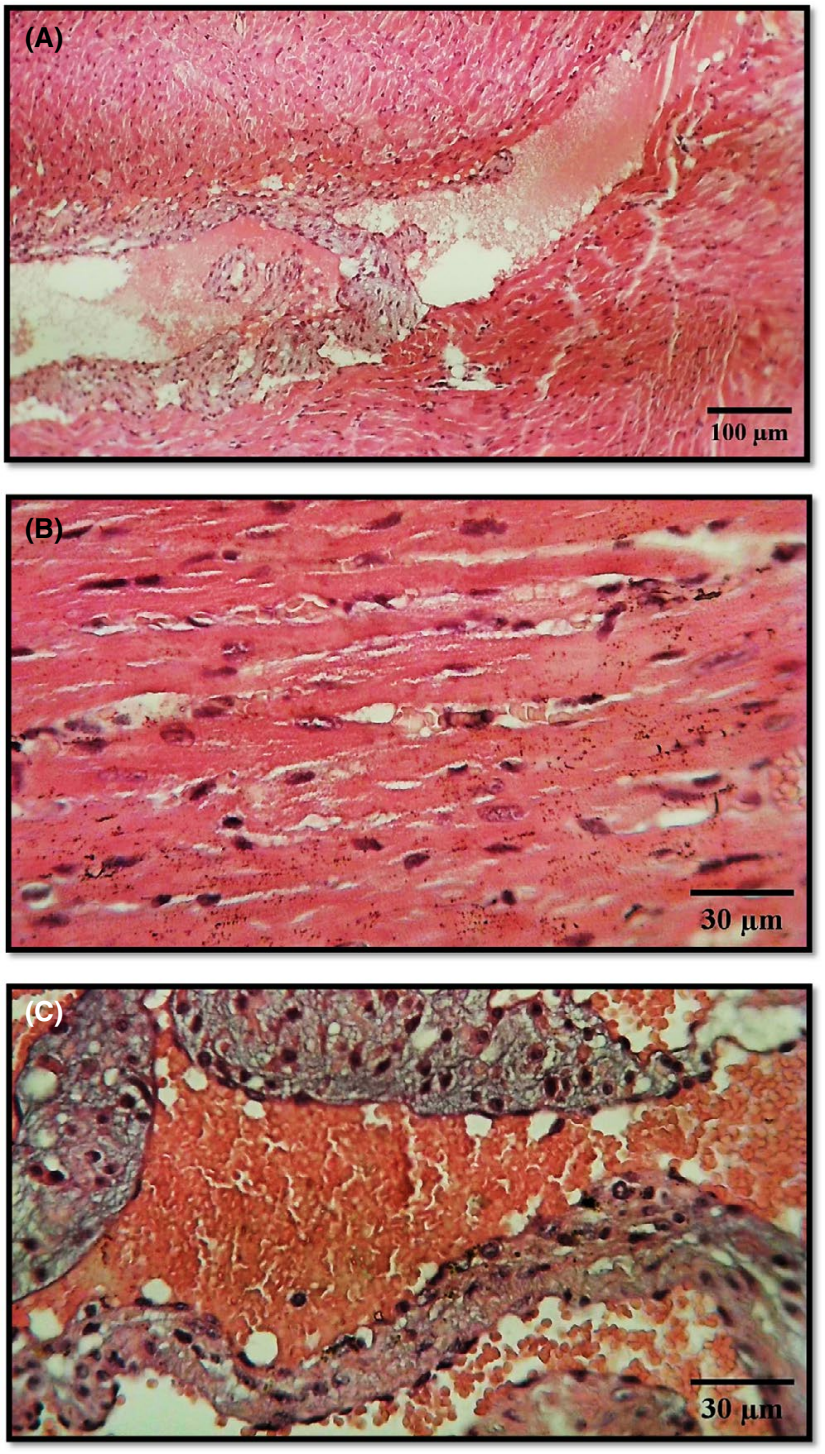
Histopathological Examination of Heart Tissue from the Control Group. (A) Overview of heart structure showing (1) myocardium, (2) heart valve, (3) atrium, (4) ventricle, and (5) endothelium (H&E, 100×). (B) Detailed view of cardiac muscle cells (1) arranged in intersecting bundles with a striated appearance, (2) featuring centrally located spherical nuclei, and (3) blood vessels exhibiting simple squamous endothelium with elongated nuclei (H&E, 400×). (C) Heart valves lined with endothelial cells (1), surrounding connective tissue containing collagen and elastic fibers (2), and visible red blood cells (RBCs) (3) (H&E, 400×).

Figure 1 presents the heart tissue structure in the control group, with Figure 1A (Hematoxylin and Eosin (H&E), 100×) showing intact myocardium, atrium, ventricle, heart valves, and endothelium, indicative of normal cardiac morphology. In Figure 1B (H&E, 400×), cardiac muscle cells (myocardium) and fibers are arranged in organized bundles, featuring a striated appearance and centrally located nuclei. Blood vessels maintain their normal structure with simple squamous endothelium. Figure 1C (H&E, 400×) further highlights the integrity of heart valves lined with endothelial cells and collagen‐rich connective tissue, with visible red blood cells (RBCs), confirming preserved tissue architecture in the control group.

#### 3.5.2 Treated group histopathology

**FIGURE 2:**
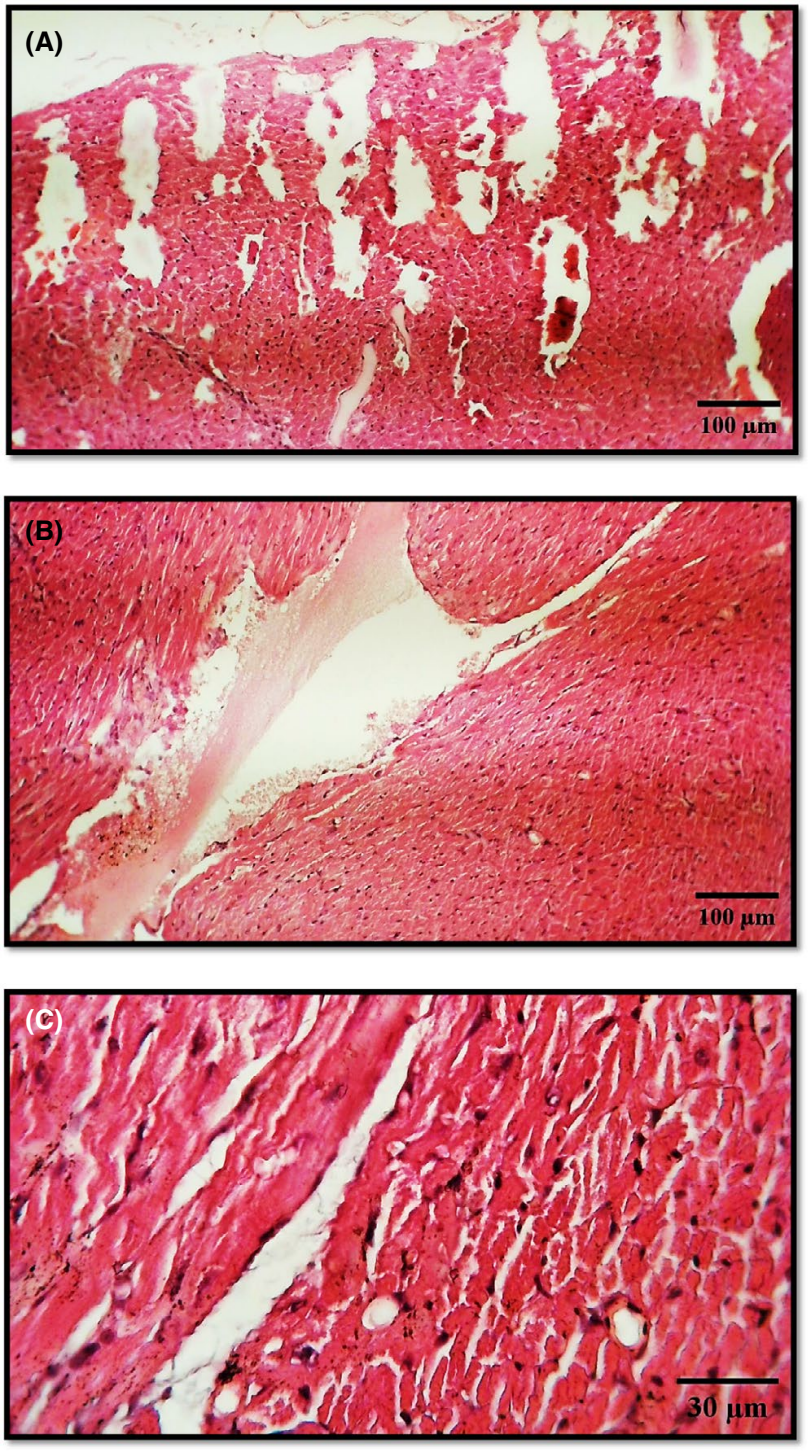
Histopathological Analysis of Cardiac Tissue from Rabbits Treated with Stanzolo (Winstrol) (20 mg/kg/day) for One Month. (A) Damage observed in (1) pericardium, (2) myocardium, and (3) blood vessels (H&E, 100×). (B) Rupture of squamous epithelial tissue in (1) endocardium, (2) myocardium, and (3) ventricle (H&E, 100×). (C) Separation of myocardial muscle fibers due to (1) tearing of heart muscle, with fibers appearing more eosinophilic than normal, and (2) dense, dark nuclei indicative of degeneration, alongside (3) damage to blood vessels characterized by wall tearing (H&E, 400×).

In contrast, Figure illustrates substantial structural damage in heart tissue sections from rabbits treated with stanozolol (Winstrol) at a dosage of 20 mg/kg/day. Figure 2A (H&E, 100X) reveals compromised pericardium, myocardium, and blood vessels, indicating severe disruption. Figure 2B (H&E, 100×) shows ruptured squamous epithelial tissue within the endocardium, with damage extending to the myocardium and ventricular tissue. Figure 2C (H&E, 400×) displays pronounced myocardial fiber separation, with fibers appearing deeply eosinophilic and nuclei showing dense chromatin, suggestive of cell degeneration. Additionally, extensive tearing in the blood vessel walls points to vascular compromise, emphasizing the detrimental effect of stanozolol on cardiac tissue integrity and function.

#### 3.5.3 Relative heart weight and collagen analysis

In the control group, the relative heart weight was 3.5 ± 0.2 g/kg, which significantly increased to 5.0 ± 0.3 g/kg in the Stanozolol‐only group (*p* < 0.001). Allicin treatment reduced this value to 4.0 ± 0.2 g/kg (*p* = 0.01). Similarly, collagen deposition was 5 ± 1% in the control group, rising to 15% ± 2% with Stanozolol administration (*p* < 0.001) but decreasing to 8% ± 1% following Allicin treatment (*p* = 0.005) (Table 6).

**TABLE 6:** Cardiac morphometric and collagen analysis.

Parameter	Control group	Stanozolol‐only group	Stanozolol + Allicin group	*p*‐Value (control vs. stanozolol)	*p*‐Value (stanozolol vs. allicin)
Relative heart weight (g/kg)	3.5 ± 0.2	5.0 ± 0.3	4.0 ± 0.2	<0.001	0.01
Collagen deposition (%)	5 ± 1	15 ± 2	8 ± 1	<0.001	0.005

**FIGURE 3:**
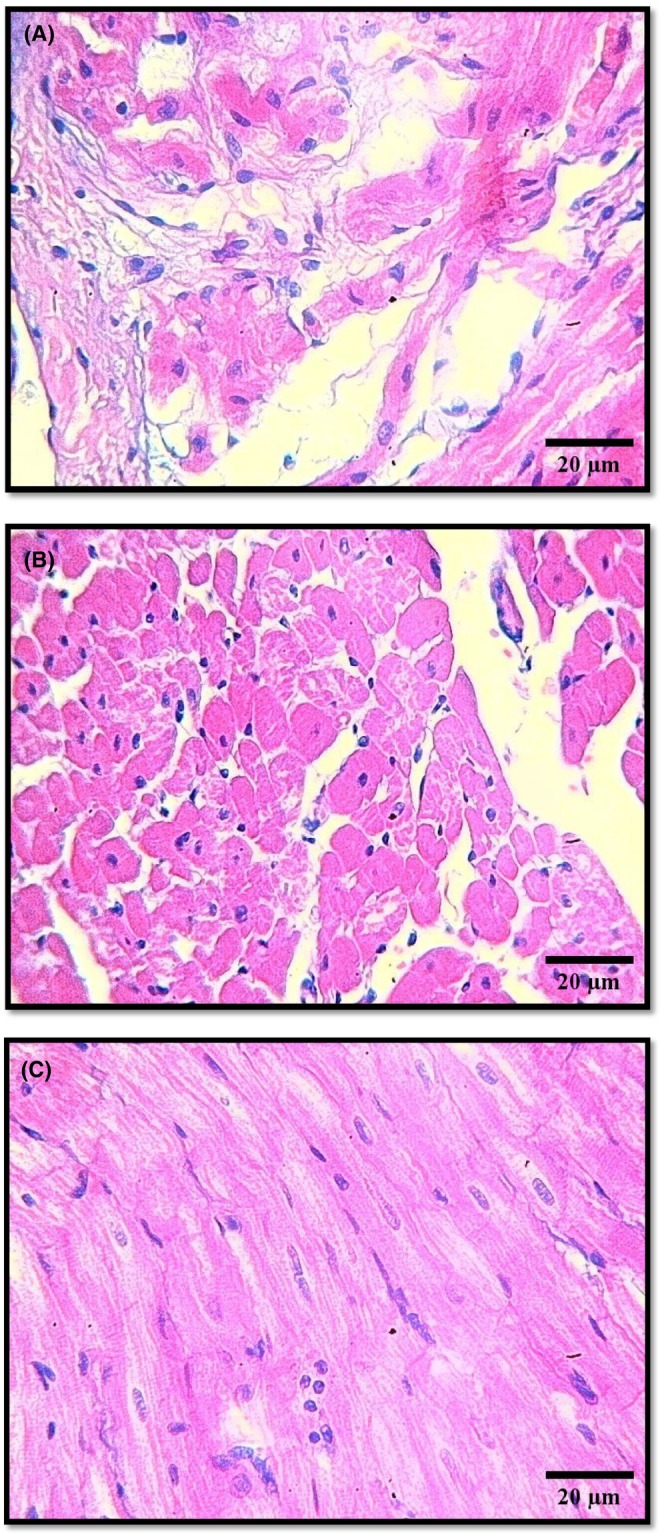
(A) showed high collagen Deposition in the treated group with Stanozolol Only (H&E, 400×). (B) showed reduced collagen Deposition with treated Stanozolol and Allicin Group (H&E, 100×) and (C) was the lowest collagen Deposition in the control group (H&E, 100×).

Analysis displayed in Figure 3 demonstrates that Winstrol (Stanozolol) along with Allicin produces different impacts on cardiac tissue collagen deposition. Figure 3A findings reveal that Stanozolol treatment as a single substance leads to enhanced collagen accumulation thus exposing serious heart tissue fibrosis and cardiotoxicity damage. The drug Stanozolol leads to pathological remodeling in heart tissue because of inflammatory conditions combined with oxidative stress. The protective effects of Allicin become apparent when collagen deposition decreases in tissue samples given Stanozolol along with Allicin when compared to samples given Stanozolol alone as shown by panel Figure 3B. By being an anti‐inflammatory and antioxidant agent Allicin demonstrates the ability to defend against the fibrotic changes caused by Stanozolol. The smallest amount of collagen exists in cardiac tissue from untreated animals who served as controls as shown by panel Figure 3C. This indicates that non‐treated tissue did not exhibit pathological remodeling at baseline. These laboratory results establish that Allicin shows value as a treatment to fight against cardiac damage from Stanozolol medication.

## 4 DISCUSSION

The present study aimed to evaluate the physiological effects of allicin in preventing cardiac damage in rabbits treated with stanozolol (Winstrol). The results indicate significant alterations in various biochemical and histopathological markers, reflecting the impact of both the drug and the treatment regimen.

### 4.1 Cardiac damage markers

The chronic treatment of stanzolo also raises the cardiospecific troponin and CK similar to other observations where anabolic steroids are also known to cause myocardial damage.^1^ Raised plasma troponin is evidence of cardiac cell injury while raised CK signifies myocardial necrosis.^15^ However, the present treatment with allacin resulted in a significant decrease in both enzymes indicating that allacin may protect myocardial tissue, which is in concordance with the reports on the cardioprotective potentials of some phytochemicals.^10^

Galectin‐3 is a biomarker associated with heart fibrosis and inflammation. The stanozolol‐only group demonstrated heightened Galectin‐3 levels relative to the control group, signifying augmented fibrotic activity. The application of Allicin therapy showed significant success in lowering Galectin‐3 levels, supporting its potential role in minimizing cardiac fibrosis.^16^ Studies have proven that allicin shields hearts from fibrosis damage by neutralizing inflammatory proteins.^17^ Stanozolol‐only exposure resulted in the increased production of GDF‐15 stress‐responsive cytokine level when compared to the control group thus demonstrating more intensive cardiac stress. The levels of GDF‐15 decreased with administration of allicin which indicates an additional protective benefit for the heart. Allicin reduces GDF‐15 levels through its mechanism which modifies inflammatory pathways and oxidative stress regulation path.^18^

### 4.2 Oxidative stress and antioxidant capacity

Research shows that MDA measured higher levels in the stanozolol group which reveals increased lipid peroxidation together with oxidative damage.^5^ Research has shown that stanozolol which is an anabolic–androgenic steroid increases MDA levels in the body thus causing elevated oxidative stress.^19^ Experiments showed that MDA decreased in the allicin‐treated group which confirms its antioxidant properties based on Nadeem et al.^20^ Research shows that allicin from garlic has proven antioxidant effects as a sulfur compound that effectively neutralizes free radicals and lowers MDA levels that reflect oxidative stress.^21^

Experimental data indicated that stanzolo treatment decreased antioxidant enzyme activity and this finding received support from Memudu and Dongo^7^ who studied antioxidant depletion due to steroid use. Glutathione levels measured in Stanozolol‐treated rats presented a significant reduction when compared to those of the control group thus showing compromised antioxidant defenses and enhanced vulnerability to oxidative damage. The group treated with stanozolol displayed considerably decreased catalase activity when contrasted to control levels which points to decreased hydrogen peroxide elimination ability. The treatment with allicin reestablished glutathione levels to similar levels as the control while simultaneously boosting catalase activity. The data shows that allicin establishes a protective antioxidant effect on the body. The consumption of allicin has been shown through recent research to raise glutathione and catalase levels which fight oxidative stress.^22^ The antioxidant enzyme activity increases under allicin administration which protects cells from oxidative damage.^23^

### 4.3 Lipid profile

Stanozolol application leads to dyslipidemia through its effects on total cholesterol levels and LDL while reducing HDL amounts. Evidence from studies indicates that stanozolol administration produces cardiovascular risk factors because it both increases cholesterol production and makes hepatic lipase activity lower HDL levels.^24^ The reported cardiovascular risk from stanozolol usage includes endothelial dysfunction and atherogenic pathway promotion according to Alyousif et al.^25^

Allicin caused extensive lipid profile changes by reducing total cholesterol levels along with LDL cholesterol but increasing the HDL cholesterol. Allicin demonstrates its lipid‐lowering properties through LDLR activity control together with PCSK9 inhibition which functions as a primary controller in cholesterol homeostasis.^26^ Furthermore demonstrated to improve antioxidant activity is allicin, which lessens oxidative stress‐induced lipid peroxidation^27^ and indirectly benefits cardiovascular health.

These results are consistent with recent studies showing allicin's ability to prevent atherosclerotic risk and treat dyslipidemia. Through its anti‐inflammatory and antioxidative qualities, allicin's benefits could go beyond lipid modification and help to protect hearts generally.^25^, ^26^

### 4.4 Inflammatory and organ damage markers

Key signals of systemic inflammation include CRP and IL‐6. Reports associating anabolic steroids to increased inflammatory responses and oxidative stress, which can aggravate cardiovascular and renal risks, match the significant increase in CRP and IL‐6 levels in the stanozolol‐only group.^4^, ^24^

Higher levels of the inflammatory index CRP and IL‐6 were also observed in the stanozolol‐treated group to the proposition that anabolic steroids could instigate infiltration of the body with inflammation.^6^ Allacin treatment significantly reduced the CRP, evidently supporting a study which noted that natural compounds can reduce inflammation via the inhibition of cytokines.^28^ Also reducing these markers with allicin co‐administration demonstrates its strong anti‐inflammatory action. By suppressing nuclear factor‐kappa B (NF‐κB), allicin has been demonstrated to block pro‐inflammatory cytokines, including IL‐6, therefore reducing inflammation.^8^, ^26^

As seen in the stanozolol‐only group, elevated Procalcitonin levels represent a pro‐inflammatory state generally linked with heart damage and systemic stress. This pro‐inflammatory reaction can aggravate myocardial damage, therefore starting an inflammatory cycle with tissue destruction. The noted drop in Procalcitonin levels following allicin treatment emphasizes its possible function in altering inflammatory pathways most likely employing NF‐κB signaling's regulation of pro‐inflammatory cytokines.^27^

AST measurements in the stanozolol‐only group demonstrate significant elevation that suggests hepatotoxicity similar to cardiac patient conditions due to either hepatic congestion or drug‐caused oxidative stress. An increase in AST levels may serve as an indication of additional organ harm that occurs due to heart failure conditions. The reduction of AST levels by allicin amplifies its antioxidant properties that protect systemic homeostasis while decreasing liver damage.^22^ Pressure injury to the heart commonly leads to renal dysfunction that presents itself through elevated Cystatin C levels because of cardiac‐renal system interactions that medical experts call cardiorenal syndrome.^25^ The renal damage from stanozolol exposure was more severe when administered alone thus leading to increased oxidative stress and renal dysfunction. In cardiology care, allicin has proven effective at reducing Cystatin C concentrations because it helps maintain antioxidant protection and leads to better renal results.^24^

Research has proven that anabolic steroids boost NF‐κB signaling pathways which results in elevated inflammatory reactions.^29^ The additional effect of anabolic steroids on iNOS and NO production creates endothelial dysfunction and oxidative stress conditions.^30^ Studies by Hernandez‐Rodriguez^31^ confirm that anabolic steroids create an inflammatory pattern by escalating TNF‐α and IL‐1β cytokines which enhance systemic inflammation. Allicin has been reported in^32^ to decrease inflammation through its action of inhibiting NF‐κB activation.^32^ The study indicates that Allicin inhibits reactive oxygen species while suppressing iNOS expression to reduce oxidative stress.^33^ According to Anarkooli et al.,^34^ Allicin shows anti‐apoptotic properties because it regulates mitochondrial pathways which leads to greater Bcl‐2 activity while reducing Caspase‐3 levels. The compound Allicin has been shown to limit pro‐inflammatory cytokine release by blocking NF‐κB signaling mechanisms which demonstrates its pathway in decreasing systemic inflammation according to.^35^

### 4.5 Histopathological analysis

Many studies document extensive cardiac toxicity through hematoxylin and eosin staining and other histological tests in rabbits treated with stanozolol while this present evaluation validates existing investigations documenting steroid‐induced cardiac tissue morphological changes.^36^ The rabbits diagnosed with allacin throughout the trial displayed enhanced favorable cardiac tissue organization demonstrating the right direction toward cardiac protection. Because of these discoveries, researchers used previous research about natural products effectively protecting cardiac structure and functionality.^37^

The administration of Stanozolol led to a major increase in heart weight because anabolic steroids trigger cardiac hypertrophy through excessive fibrotic changes and elevated myocardial workload according to studies by Schimmel et al.^38^ Allicin treatment resulted in heart weight reduction indicating myocardial protection because this compound might decrease myocardial inflammatory response and oxidative stress.^39^ Both collagen deposition levels and the role that Stanozolol‐only use plays in cardiac fibrosis are verified by research documenting anabolic steroids boost TGF‐beta (TGF‐β) signaling pathways to recreate extracellular matrix remodeling.^40^ Previous findings showed that allicin decreases collagen buildup yet at the same time the study confirms its anti‐fibrotic properties and TGF‐β suppression and downregulation of specific profibrotic genes.^41^

## 5 CONCLUSION

The present study reveals that allicin provides remarkable cardioprotective effects against stanozolol‐induced toxicity. It does this by reducing oxidative stress, suppressing inflammatory responses, and maintaining the integrity of myocardial structure. Allicin administration led to a notable decrease in biomarkers of cardiac injury (troponin, CK, Galectin‐3, GDF‐15), oxidative stress markers (MDA, glutathione, catalase), inflammatory cytokines (CRP, IL‐6, NF‐κB, TNF‐α, IL‐1β) and improved lipid profiles.

The histopathological finding was confirmed to prevent cardiac remodeling, fibrosis, and reduced collagen deposition. These findings support the potential of allicin as a protective agent for cardiovascular health in the context of anabolic steroid‐induced cardiotoxicity.

## 6 LIMITATION OF THE STUDY

One of the limitations of this study is the restricted number of parameters analyzed. The inclusion of additional parameters could have provided a more comprehensive understanding of the Physiological effect of allicin to prevent cardiac damage.

## AUTHOR CONTRIBUTIONS

**Mohammed Hayder Asker:** Conceptualization; data curation; formal analysis. **Noor AL‐Huda Salah AL‐Zuhairy:** Formal analysis; funding acquisition; methodology. **Wassan Mhammed Husain:** Software; supervision; validation; visualization. **Mustafa Riyadh Abdullah:** Resources; software; writing – original draft; writing – review and editing.

## FUNDING INFORMATION

The research and preparation of this manuscript were conducted without receiving any external funding or financial support. Every facet of the research, encompassing the gathering, processing, evaluation, and composition of the text, was carried out independently by the authors.

## CONFLICT OF INTEREST STATEMENT

The authors of this publication affirm that they have no conflicting interests that could skew how the study findings are interpreted or presented. There are no personal or financial ties to any other people or organizations that may unintentionally affect the research described in this paper.

## ETHICS STATEMENT

Animal procedures approved by the Institutional Animal Care and Use Committee (IACUC) at Mustansiriyah University (Approval No. 42, 1/9/2024).

## CONSENT TO PARTICIPATE

Informed consent was obtained from all individual participants included in the study.

## Data Availability

The datasets generated during and/or analyzed during the current study are available from the corresponding author on reasonable request.
